# New Dental Colleges

**Published:** 1891-09

**Authors:** 


### New Dental Colleges.

How many new dental colleges have been organized in this country, and will enter upon a course of work this fall, we do not know; but we have received the announcements of several. Of these, there is the Dental Department of the Homœopathic Hospital College, of Cleveland, Ohio, fully organized and equipped, and will begin its first regular session on the 23rd inst. It has a full faculty, of which W. H. Whitslar, M.D., D.D.S., of Youngstown, Ohio, is Dean, and Professor of Principles and Practice of Dental Science, Dental Pathology and Embryology. George H. Wilson, D.D.S., of Painsville, Ohio, is Superintendent of Dental Infirmary. In the announcement it is stated that the college has adopted the requirements agreed upon by the National Association of Dental Faculties of the United States. The term is to be six months, and three full courses of lectures will be required for graduation. The requisites for admission are a degree in letters or science, a diploma from a high school or academy, a teacher's certificate, or to pass a creditable examination in orthography, penmanship composition, arithmetic, English grammar and United States history. It will be seen from this statement that the institution will start upon a good basis, and if it does good, faithful, efficient work, it should be recognized as a desirable acquisition to the educational resources of the profession in this country.

We have also received an announcement of the Dental Department of the Cincinnati College of Medicine and Surgery, Cincinnati, O. This has also been organized in connection with the medical college as just stated. G. S. Junkerman, M.D., D.D.S., is Dean of the Faculty, and Professor of Operative Dentistry and Oral Surgery. Charles H. Martin, D.D.S., is the Secretary of the Faculty, Professor of Dental Materia Medica and Dental Pathology. The announcement contains the following: "The Dental Department of the Cincinnati College of Medicine and Surgery will conform to all the rules and regulations, and all the requirements of The National Association of Dental Faculties." This school also proposes a six months term. The various branches of the course, except those strictly special, are taught by the various professors of the medical faculty.

We have just received information, through a private source, that a dental department is organized in connection with a medical college in Detroit, and that it also will begin its first course this fall. We are not advised fully as to its details, and so are not just now able to specify. This is an enterprise, however, that has been talked of for two or three years, and the organization has been a foregone conclusion for some time. If it is put upon a sound basis, and proposes thorough work in the preparation of its students, and is faithful to the best interests of the profession, who shall say nay? Most of the dental colleges or departments that have been organized within the last four or five years have been in connection with, and are fostered by medical colleges; this is a natural result, perhaps from the recognition of dentistry as a specialty of medicine that has been so pronouncedly made within the last few years. In nearly every instance where these departments have been organized it has been upon the solicitation, or at least, suggestion on the part of medical faculties. In this fact is shown a great contrast between the present time and the time when Drs. Chapin A. Harris and H. H. Hayden sought to have a dental department organized in connection with a medical college in Baltimore, when the proposition was received with little short of actual contempt; truly the world moves.

